# Supplementary material for: A double role of the Gal80 N terminus in activation of transcription by Gal4p
Source: Life Sci Alliance. 2020 Oct 9;3(12):e202000665. doi: 10.26508/lsa.202000665 (PMC7556753; doi:10.26508/lsa.202000665)
Supplement: Supplementary file 1 [file LSA-2020-00665_TableS1.docx]

**Supplementary Table S1: Overview of yeast strains**

| Strain | Genotype | Species | Origin |
| --- | --- | --- | --- |
| JA6/G80-KR56A | *gal80-K5A, R6A* | *K. lactis* | This work |
| JA6/G80M | *GAL80* | *K. lactis* | This work |
| JA6/G80-SV40 | *SV40-NLS-GAL80* | *K. lactis* | This work |
| JA6/G80-SVKR | *SV40-NLS-gal80-K5A, R6A* | *K. lactis* | This work |
|  |  |  |  |
| FI4 sin4∆ ScGAL80 | *ScGAL80* | *S. cerevisiae* | This work |
| FI4 sin4∆ ScGAL80KR56A | *Scgal80-K5A, R6A* | *S. cerevisiae* | This work |
