# Supplementary material for: A double role of the Gal80 N terminus in activation of transcription by Gal4p
Source: Life Sci Alliance. 2020 Oct 9;3(12):e202000665. doi: 10.26508/lsa.202000665 (PMC7556753; doi:10.26508/lsa.202000665)
Supplement: Supplementary file 2 [file LSA-2020-00665_TableS2.docx]

**Supplementary Table S2: Overview of used plasmids**

| **Plasmid** | **Description** | **Origin** |
| --- | --- | --- |
| pAG80 | *KlGAL80* with *ScADH1*-promoter | Zenke et al., 1999 |
| pAG80KR56A | *Klgal80-K5A, R6A* with *ScADH1*-promoter | This work |
| pCGal1HA | *KlGAL1-(HA)_3_* | This work |
| pCGFPAG1 | *GFP-KlGAL1* with *ScADH1*-promoter | Anders et al., 2006 |
| pCGFPAG1-ura3Δ | *GFP-KlGAL1* with *ScADH1*-promoter, *Scura3* | Anders et al., 2006 |
| pCNLSGal1HA | *SV40-NLS-KlGAL1-(HA)_6_* | This work |
| pEAG80 | multi copy plasmid, *KlGAL80* with *ScADH1*-promoter | Zenke et al., 1999 |
| pEAG80KR56A | multi copy plasmid, *Klgal80-K5A, R6A* with *ScADH1*-promoter | This work |
| pEAG80-KR56A-SV40 | multi copy plasmid, *SV40-NLS-gal80-K5A, R6A* with *ScADH1*-promoter | This work |
| pEAG80S2 | multi copy plasmid, *Klgal80-S2* with *ScADH1*-promoter | Zenke et al., 1999 |
| pEG80WTGFPct | multi copy plasmid, *KlGAL80-GFP* with *ScADH1*-promoter | This work |
| pEgal80NLS1 | multi copy plasmid, *GFP-Klgal80-K5A, R6A* with *ScADH1*-promoter | This work |
| pEgal80NLS1C1 | multi copy plasmid, *GFP-Klgal80-K5A, R6A-C1* with *ScADH1*-promoter | This work |
| pEgal80NLS1GFPct | multi copy plasmid, *KlGAL80-K5A, R6A-GFP* with *ScADH1*-promoter | This work |
| pEGFP-Kl56-ScG80 | multi copy plasmid, *GFP-Scgal80- K5A, R6A* (coding for GFP-ScGal80p with KlGal80p-K5A/R6A N-terminus aa 1- 16) with *ScADH1*-promoter | This work |
| pEGFP-KlNT-ScG80 | multi copy plasmid, *GFP-Scgal80-KlNT* (coding for GFP-ScGal80p with KlGal80p N-terminus aa 1- 16) with *ScADH1*-promoter | This work |
| pEGFPScG80-S8K | multi copy plasmid, *GFP-Scgal80-S8K* with *ScADH1*-promoter | This work |
| pEQRS80 | multi copy plasmid, *GFP-KlGAL80* with *ScADH1-promoter* | Hager, 2003 |
| pEQRS80DC1 | multi copy plasmid, *GFP-Klgal80-DC1* (aa 40-457) | This work |
| pEScG80 | multi copy plasmid, *GFP-ScGAL80* with *ScADH1-promoter* | This work |
| pEScG8036 | multi copy plasmid, Scgal80-36 (aa 1-36) with *ScADH1-promoter* | This work |
| pETIHG80 | expression plasmid, *IHKlGAL80* (coding for internal His_6_-tagged Gal80p) | Anders et al., 2006 |
| pETIHG80KR56A | expression plasmid, *IHKlgal80-K5A, R6A* (coding for internal His_6_-tagged Gal80-K5A/R6Ap) | This work |
| pETNHG80 | expression plasmid, *NHKlGAL80* (coding for n-terminal His_6_-tagged Gal80p) | Anders et al., 2006 |
| pETNHG80KR56A | expression plasmid, *NHKlgal80-K5A, R6A* (coding for n-terminal His_6_-tagged Gal80-K5A/R6Ap) | This work |
| pGSTGal1 | *GST-GAL1* | Zenke et al., 1999 |
| pI80 | integration plasmid, *KlGAL80* with upstream und  downtream sequences | Zachariae and Breunig, 1993 |
| pI80KR56A | integration plasmid, *Klgal80-K5A, R6A* with upstream und downtream sequences | This work |
| pI80KR56ASV40 | integration plasmid, *SV40-NLS-gal80-K5A, R6A* with upstream und downtream sequences | This work |
| pI80Myc | integration plasmid, *KlGAL80*-c-myc with upstream und downtream sequences | This work |
| pKlGal80KR56A | *Klgal80-K5A, R6A* | This work |
| pScGal80 | *ScGAL80* | Breunig lab |
| pYM5 | c-myc-tag | Knop et al., 1999 |
