# Supplementary material for: A double role of the Gal80 N terminus in activation of transcription by Gal4p
Source: Life Sci Alliance. 2020 Oct 9;3(12):e202000665. doi: 10.26508/lsa.202000665 (PMC7556753; doi:10.26508/lsa.202000665)
Supplement: Supplementary file 3 [file LSA-2020-00665_TableS3.docx]

**Supplementary Table S3: Overview of DNA oligonucleotide sequences used for cloning**

| Primer name | Sequence |
| --- | --- |
| ADH1-G80-inFu-FW | 5`- TCGGCCGCTCTAGAAGATCCGGGATCGAAGAAAT -3` |
| ADH1-G80-inFu-RV | 5`- GAAGGGAGTGTCCAGGGTGGTGATTCCTGACTG -3` |
| Amp_DS | 5`- TTACCAATGCTTAATCAGTGAGGCACCTATCTCAGCGAT -3` |
| C1MluIKlG80Fw | 5`-ATGGGTGCTCCACCAAAAAAAAAAAGAAAAGTTGCTAACAATA  ACGCTGCTTCCAA -3` |
| C1SmiIKlG80Bw | 5`- GCGGATTTAAATGGCTAAGAAATGCGT -3` |
| C2MluIKlG80FwNeu | 5`-TAATACGCGTAAGACGCATTTCTT -3` |
| G80-KR56A-InFu-FW | 5`- CTGGACACTCCCTTCCAT -3` |
| G80-KR56A-inFu-RV | 5`- CCGATTTAAAGATTTAAATGCGCAACAAGTCTTTAA -3` |
| GFPFw | 5`- GGGATTACACATGGCATGGATGAACT -3` |
| Integration_pIG80_3 | 5`- CAGCCACAAGAATCTCACCA -3` |
| KlG80_KR56A_NT15AS_fw | 5`- CAAAACGCGTAACAATAACGCTGCTTCCAAATTATCCACGGTG  CCATCGAGTAGACCCATAAGAGTCGGATTCGTCG -3` |
| KlG80_rv | 5`- GCCATAACGGATTCCCGATTTAAAGA -3` |
| KlG80KR56A_fw | 5`- CATGAACAATAACGCTGCTTCCAAATTATCCACGGTGCC -3` |
| KlG80NT_15AS_fw | 5`- CAAAACGCGTAACAATAACAAACGGTCC -3` |
| KlG80SmiIBw | 5`- ACGTAAGCAAGCCATAACGGATTCC -3` |
| KlGAL80-422C | 5`- CTCTTCTGCCTGTTGTACGC -3` |
| KlGal80NLS1CO | 5`- GACGGCATCATGAACAATAACGCTGCTTCCAAATTATCCACGG  TGCC -3` |
| MluATGWTG80fw | 5`- AGGCTACGCGTATGAACAATAACAAACGGTCCAAATTATC -3` |
| MluIScGal80fw | 5`- CAAAACGCGTGACTACAACAAGAGATCTT -3` |
| MycTagBw | 5`- TGGATACATAGATTTGAAGTTTATTTACTAGACTCTAGATGA  TCCGTTCA -3` |
| ScG80_36_Smi_Bw | 5`- GCGGATTTAAATTAATGTGTCTTGATTG -3` |
| ScG80_K8_fw | 5`- CAAAACGCGTGACTACAACAAGAGATCTAAGGTCTCAACC  G -3` |
| ScGal80NESMuta | 5`- TAACCTGTGTAATATCGCAGCATCCATCGCGGTGGGAAAG  CCTTCG -3` |
| SV40NLS_KR56A_fw | 5`- ATGGGTGCTCCACCAAAAAAAAAAAGAAAAGTTGCTAACAA  TAACGCTGCTTCCAA -3` |
| SV40NLSKlG80_bw | 5`- AGCAACTTTTCTTTTTTTTTTTGGTGGAGCACCCATGATGCC  GTCCTGCCGAGA -3` |
| SwaIScGal80bw | 5`- CGACGGATTTAAATTTATAAACTATAATGCG -3` |
| TagFw | 5`- TTGACGTGTCGAAAATAATGATACGTACGCTGCAGGTCGA  CGG -3` |
