# Supplementary material for: A double role of the Gal80 N terminus in activation of transcription by Gal4p
Source: Life Sci Alliance. 2020 Oct 9;3(12):e202000665. doi: 10.26508/lsa.202000665 (PMC7556753; doi:10.26508/lsa.202000665)
Supplement: Supplementary file 4 [file LSA-2020-00665_TableS4.docx]

**Supplementary Table S4: Overview of oligonucleotides used for KlGal80 fragment construction**

| Fragment^1^ | Included residues^2^ | Number of residues^2^ | First Primer^3^ | Second Primer^3^ |
| --- | --- | --- | --- | --- |
| A1 | 2-166 | 165 | GFPfw  5`-ATTACACATGGCATGGATGAA  CT-3` | A1KlG80SmiIBw  5`-ATTTAAATTGATTAACTCTTTG  GCCCG-3` |
| A2 | 2-332 | 331 | GFPfw  5`-ATTACACATGGCATGGATGAA  CT-3` | A2KlG80SmiIBw  5`-ATTTAAATGCTGCTACCGTTC  CCATTC-3` |
| A3 | 307-457 | 151 | MluIA3KlG80Fw  5`-ACGCGTGAGGGTGATGCA-3` | KlG80SmiIBw  5`-ACGTAAGCAAGCCATAACGG  ATTCC-3` |
| B1 | 151-346 | 196 | B1MluIKlG80Fw  5`-ATTATACGCGTCTCCAAGGACG  TAAAT-3` | B1SmiIKlG80BwNeu  5`-GGCGCATTTAAATCTTGTCT  TTTAT-3` |
| B2 | 167-240 | 73 | B2MluIKlG80FwNeu  5`-AATTACGCGTAGCGAAGGTTG  T-3` | B2SmiIKlG80Bw  5`-GGCGCATTTAAATAGTTGGGAT  ATTGTTTG-3` |
| B3 | 231-332 | 102 | B3MluIKlG80Fw  5`-AATATACGCGTAATGCGATGAT  CTCAAAC-3` | B3SmiIKlG80Bw  5`-AATAGATTTAAATGCTGCTACC  GTTCCCAT-3` |
| C1 | 2-39 | 38 | C1MluIKlG80Fw  5`-GCGAACGCGTAACAATAACAAA  CGGTC-3` | C1SmiIKlG80Bw  5`-GCGGATTTAAATGGCTAAGA  AATGCGT-3` |
| C2 | 34-72 | 39 | C2MluIKlG80FwNeu  5`-TAATACGCGTAAGACGCATTT  CTT-3` | C2SmiIKlG80Bw  5`-GCCGATTTAAATTAGCATGTTT  CAATTGC-3` |
| C3 | 67-104 | 37 | C3MluIKlG80FwNeu  5`-AACTACGCGTTTGCAATTGAA  AC-3` | C3SmiIKlG80Bw  5`-GCCCATTTAAATCTTGACCACC  TC-3` |
| C4 | 101-139 | 39 | C4MluIKlG80Fw  5`-TAATACGCGTGAGGTGGTCAA  GAA-3` | C4SmiIKlG80Bw  5`-GGCGATTTAAATGAGATATCGA  ATACAACT-3` |
| C5 | 134-166 | 33 | C5MluIKlG80FwNeu  5`-CAGCACGCGTGAGTTGTATT-3` | C5SmiIKlG80BwNeu  5`-CGGGATTTAAATGATTAACTCT  TTG-3` |

^1^The fragment IDs are identical to those in Fig. S1.

^2^Residues are given as positions and number of amino acids in full length KlGal80 protein (without GFP).

^3^Plasmid pEQRS80 (Hager, 2003) containing the reading frame for a full-length KlGal80-GFP fusion protein was used as template for PCR amplification of *KlGAL80* subfragments with the listed primer pairs. The PCR products and the pEQRS80 vector were cleaved with *Mlu*I and *Smi*I and ligated.
